# Adenoid cystic carcinoma of the thymus gland

**DOI:** 10.1186/s13019-023-02432-w

**Published:** 2023-11-09

**Authors:** Mohamed Shatila, Hanan Hemead, Nishanth Murukesh, Philippe Taniere, Caroline Russell, Ashvini Menon, Akshay J. Patel

**Affiliations:** 1 Department of Thoracic Surgery, University Hospitals Birmingham NHS Trust, Birmingham, UK; 2 Department of Cardiothoracic Surgery, Faculty of Medicine, Alexandria University Hospital, Alexandria, Egypt; 3 Department of Medical Oncology, Worcestershire Royal Hospitals NHS Trust, Worcester, UK; 4 Department of Cellular Histopathology, University Hospitals Birmingham, NHS Trust, Birmingham, UK; 5 Institute of Immunology and Immunotherapy, University of Birmingham, Vincent Drive, Edgbaston, B15 2TT UK

**Keywords:** Adenoid cystic carcinoma (ACC), Thymus, Anterior mediastinal mass, Epithelial tumours, Thymic carcinoma with adenoid cystic carcinoma-like features (TCACC)

## Abstract

**Background:**

Thymic carcinomas are rare and aggressive tumours. They constitute a heterogeneous group of tumours with various histological patterns and subtypes resembling epithelial tumours arising from other organs.

Case presentation.

We hereby represent a case of primary thymic carcinoma with adenoid cystic carcinoma-like features (TCACC) which is an extremely rare variant of thymic adenocarcinoma. To date and to the best of our knowledge, there are nine reported cases in literature and ours is the tenth. Our case was treated surgically but the implementation of adjuvant chemoradiotherapy has been reported in few of the published cases.

**Conclusions:**

TCACC constitutes a rare entity of thymic adenocarcinoma with limited available literature. The current data is derived from few case reports and case series. The histological overlap of these tumours and primary ACC of salivary glands poses a diagnostic challenge. Radiological investigations, immunohistochemical phenotyping and genetic analysis are crucial in establishing the diagnosis.

## Introduction

Thymic carcinomas are rare tumours with several clinic-pathological variants. They pose a diagnostic challenge due to the different histological presentations and rare incidence, with cases having only been described through very small retrospective series. The fifth version of WHO classification of thymic tumours describes four entities of adenocarcinomas; papillary, adenoid cystic carcinoma like features, enteric and non-otherwise specified. Adenoid cystic carcinoma (ACC) is a rare primary tumour of salivary glands which infrequently arise in other organs such as breast and lungs. Thymic carcinoma with adenoid cystic carcinoma-like features (TCACC) is exceedingly rare. Initially these tumours were labelled as adenoid cystic carcinoma (ACC) of thymus gland, however; due to the lack of immunohistochemical features of ACC, they are now called thymic carcinoma with adenoid cystic carcinoma-like features. Histological features of TCACC mimics the adenoid cystic carcinoma of salivary gland. Immunohistochemical staining is pivotal in establishing the diagnosis [1].

In 2007, Di Tommaso et al. reported the first case series of TCACC described in four patients. The clinicopathological behaviour of this subtype and the long-term survival is not fully understood given the paucity of cases [2].

## Case presentation

A fifty-seven-year-old Caucasian male presented with progressive exertional dyspnoea and occasional sharp left sided chest pain over a two-week period. He is otherwise a fit and active with no significant past medical history.

**Fig. 1:**
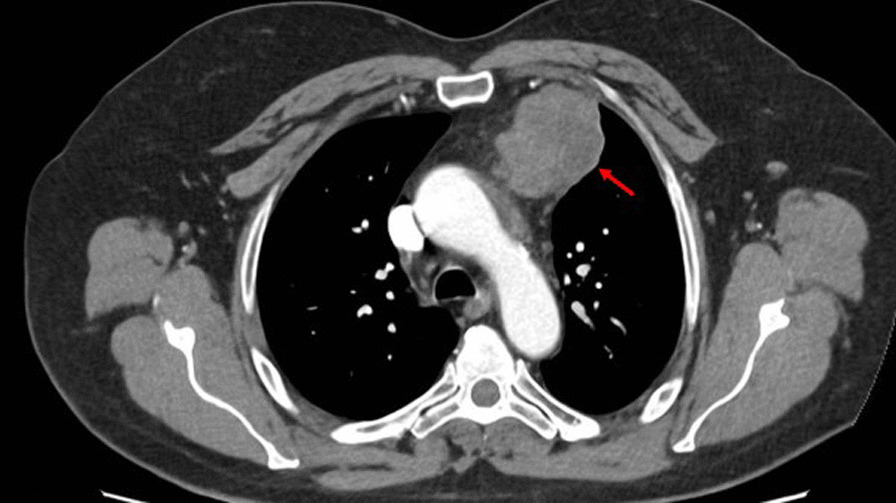
Axial slice of contrast-enhanced CT slice of the thorax demonstrating the mediastinal mass (red arrow)

**Fig. 2:**
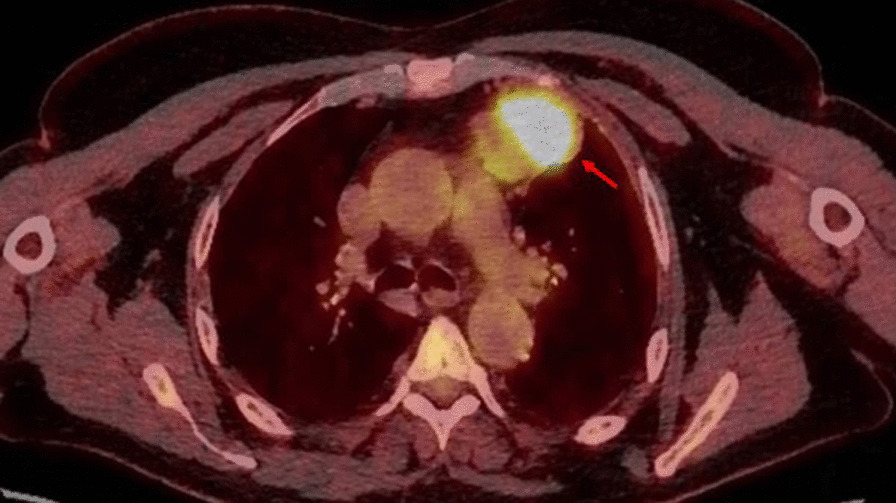
Axial slice of PET-CT demonstrating the PET avid mediastinal mass (red arrow)

Clinical examination and routine investigations were unremarkable. Chest-x ray revealed a left hilar soft tissue density which was suggestive for lymphadenopathy. Computed tomography (CT) of the chest showed a lobulated anterior mediastinal mass measuring 5.8 × 4.9 cm, abutting the pericardium with no visible fat plane and bulging into the medial aspect of the left lung without signs of infiltration (Fig. 1). Differential diagnoses included a thymic tumour and lymphoma. The latter was radiologically and clinically less favourable after discussion with haematologists and normal investigations and blood tumour marker profile. However, the CT guided biopsy showed cores of an adenoid cystic carcinoma-like tumour presenting either a primary thymic adenocarcinoma with adenoid cystic pattern or metastasis from salivary gland origin. Positron emission tomography (PET) scan (Fig. 2) showed a highly avid anterior mediastinal mass with an SUV max of 10.4 and no tracer uptake in the salivary glands or elsewhere ruling out metastasis from salivary glands. There was no medical history of salivary gland neoplasms in this patient and a CT of the head/neck region showed no evidence of malignancy.

**Fig. 3:**
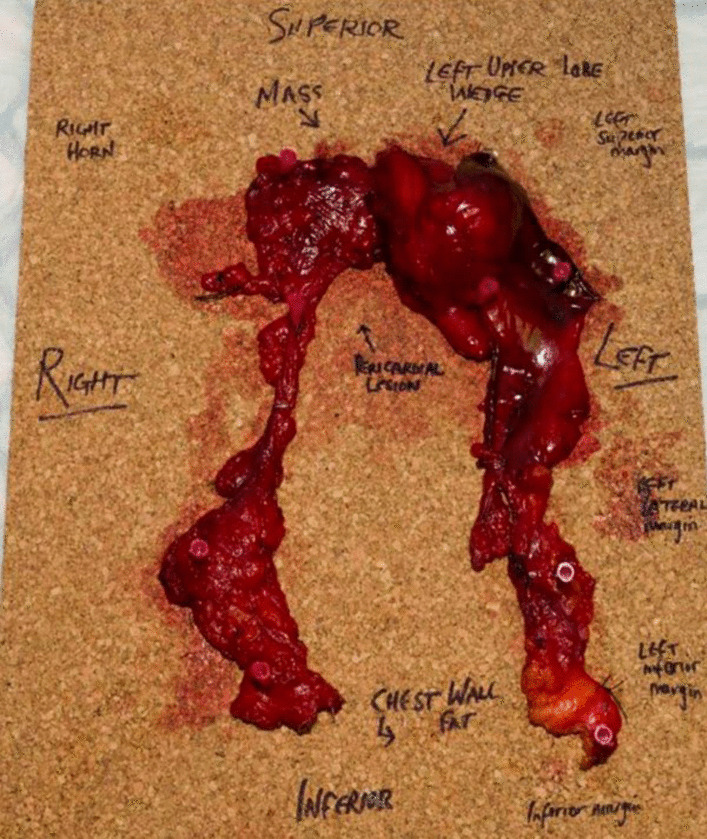
Macroscopic appearance of fully resected specimen on cork board post operatively with labelling of the relevant anatomical features and margins

The patient was thence referred for discussion in the thymoma multi-disciplinary meeting (MDT) which recommended surgical resection. Pericardial and chest wall invasion was excluded with magnetic resonance imaging (MRI). The mass was approached through median sternotomy. An encapsulated lesion lying towards the left side was seen involving the left upper lobe of the lung and pericardium with doubtful invasion of the pleura. The tumour was completely resected including an 85 X 20 mm left upper wedge and 40 mm pericardial edge (Fig. 3). The adjacent parietal pleura was sent for frozen section which showed no atypia or malignancy but rather an inflamed pleura.

**Fig. 4:**
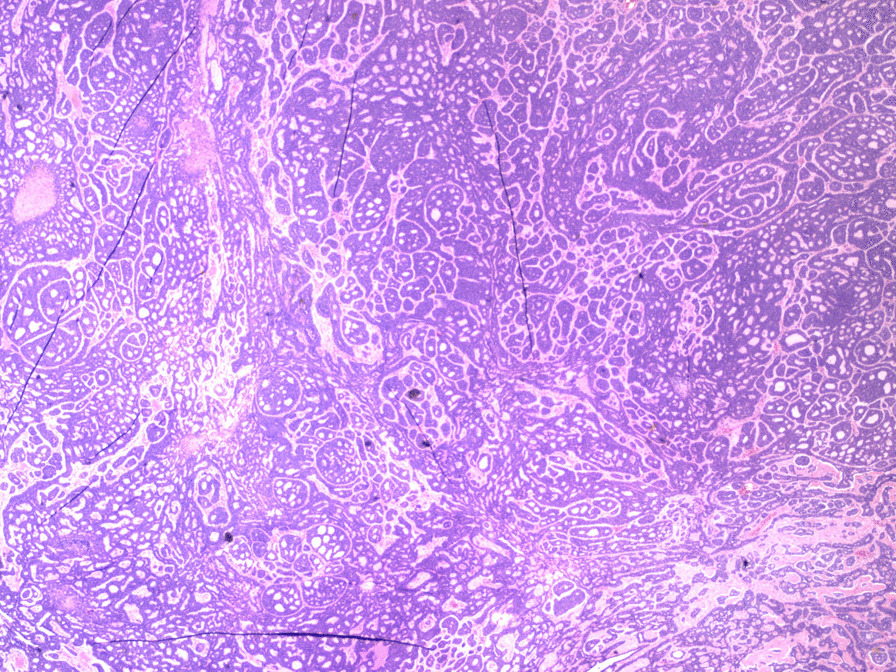
Histological specimen slice of the resected mass

**Fig. 5:**
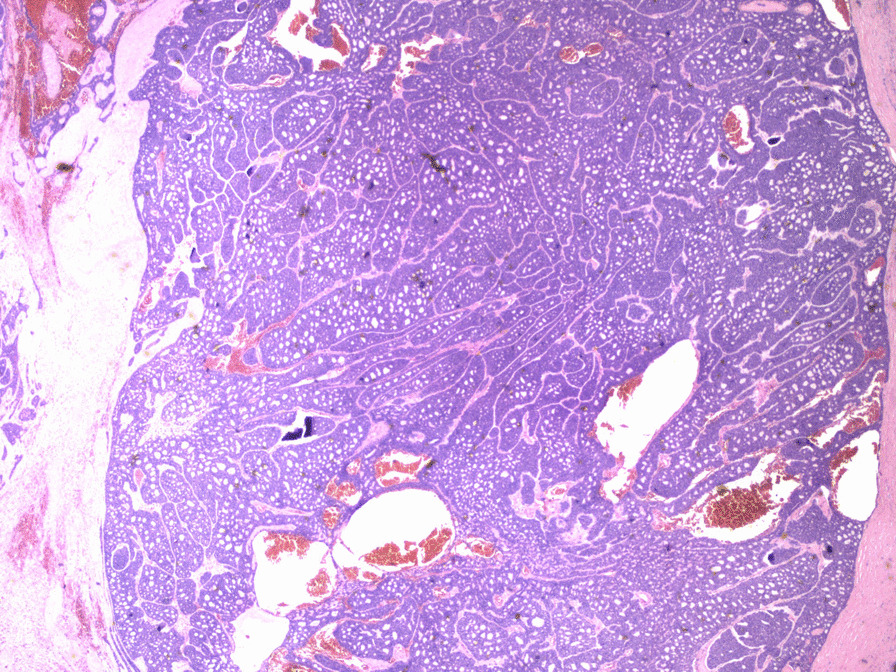
Histological specimen slice of the resected mass

Macroscopically, the mass measured 50 × 25 × 58 mm without evidence of capsular breach. Microscopically, a predominant cribriform architecture with both solid and cystic degenerated areas were seen. Mitotic figures were infrequent with frequent foci of tumour necrosis. A panel of immunohistochemistry markers was conducted. The tumour cells were strongly positive for CD117, CK7, with patchy positivity for CK19. The immunohistochemistry confirmed the presence of epithelial and myoepithelial components (p63, p40 and CK5/6 positive). Figures 4 and 5 show histological slices of the specimen.

**Fig. 6:**
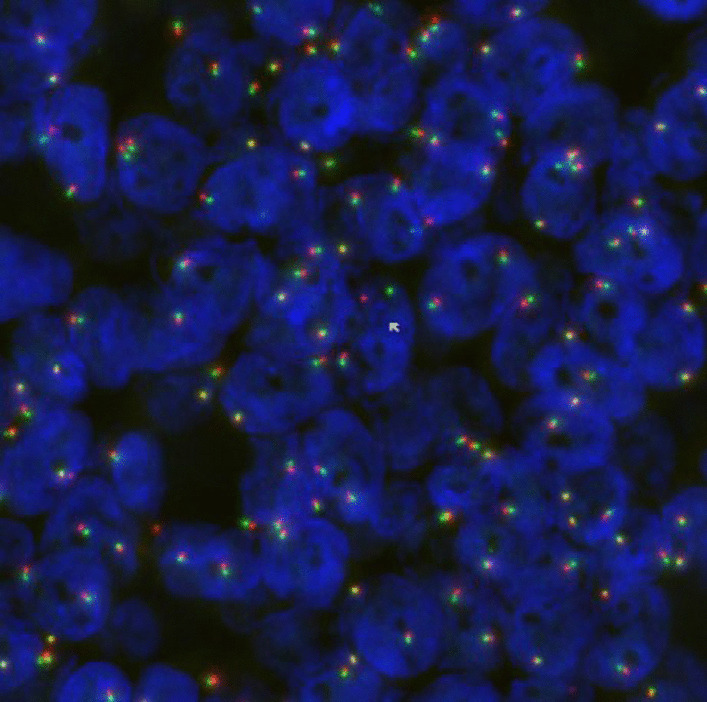
MYB rearrangement visual

Taking into consideration the morphology, the positive expression of CD117 and the presence of epithelial and myoepithelial components, metastasis from adenoid cystic carcinoma was favoured over primary thymic carcinoma with adenoid cystic-like features. A true primary adenoid cystic carcinoma is very unusual and careful assessment was performed to definitively exclude the possibility of metastasis. Further testing was done on the tumour for an MYB gene rearrangement. Results showed no clear evidence of rearrangement at 6q23.2-q23.3 (Fig. 6).

Grading of the tumour was rather challenging. The proportion of solid areas present was less than 30% and therefore it was considered as a low-grade lesion, which is the same classification as for primary salivary tumours. There was however patchy necrosis with lymphatic and venous invasion which could indicate a high degree of aggression. The tumour was staged based on the TNM 8th edition as a pT1bN0Mx, R0 with lymphatic and venous invasion.

The morphology between ACC and TCACC is different and the IHC is different both in terms of markers expressed and pattern of expression. Periodic Acid Schiff was helpful (material positive in ACC but not TCACC). This tumour was unusual in that the amount of necrosis and the vascular invasion was patchy hence the need to exclude a primary elsewhere.

## Comment

ACC of the salivary glands account for 1% of all head and neck cancers and 10% of all salivary gland tumours. Histologically, these tumours are characterized by basaloid cells with epithelial and myoepithelial elements. The myoepithelial cells of pseudocysts can be identified by various markers such as the S100 protein, muscle actin, p63, CD117 (receptor tyrosine kinase c-Kit) and vimentin [3]. The translocation of the MYB gene is characteristic of ACC. This close resemblance makes differentiating between ACC of salivary glands and TCACC quite challenging [4]. With the help of a full body PET/CT, ruling out other primaries, and lack of MYB gene rearrangement, the diagnosis of TCACC can be more reliable.

To the best of our knowledge, only 9 cases have been reported in literature (summarised in Table 1. below). The ages of all 10 patients, including ours, ranged from 37 to 77 years. Only one case reported by Kanazaki R et al. showed evidence of distant metastasis. In this case, the entire thymus gland including the tumour, manubrium, bilateral proximal clavicles and the right fifth rib were removed with a metastatic nodule resected from the right middle lobe [5].

**Table 1:** A summary of the reported cases of thymic adenoid cystic carcinoma in the literature

#	Author	Year	Age/sex	Size (cm)	Metastasis	Treatment	CD117 expression
1	Di Tommaso	2007	65/M	5	No	S	Positive
2	Di Tommaso	2007	63/F	2.5 × 2	No	S	Negative
3	Di Tommaso	2007	69/M	13 × 10	No	S, R	Negative
4	Di Tommaso	2007	77/M	13 × 10	No	S	Negative
5	Coulibaly	2008	37/F	Large	No	S, C, R	Not documented
6	Banki F	2010	65/F	14 × 8.8	No	S	Not documented
7	Kanzaki R	2011	66/M	4.9 × 4.4	Yes	S	Negative
8	Rampisela D	2015	47/M	15 × 12	No	S, R	Not documented
9	Mai-Qing Yang	2019	38/M	5 × 3.5	No	S	Positive
*10*	*Present Case (Shatila MS *et al.*)*	*2023*	*57/M*	*5.8* × *4.9*	*No*	*S*	Positive

*F* female; *M* male; *C* chemotherapy; *R* radiotherapy; *S* surgery

All reported cases [6–9], including ours, were treated surgically with two patients requiring adjuvant radiotherapy and only one patient showed evidence of histopathological perineural and vascular invasion along with pericardial invasion. The latter patient was offered both adjuvant chemotherapy and radiotherapy [6]. With respect to survival, one patient died of myeloid leukaemia after 5 years of surgery and two patients were lost to follow up.

In our case, the patient was treated surgically with clear margins, achieving an R0 resection. No adjuvant treatment was indicated. Currently, the patient has completed his first 3-month follow-up with good post operative recovery. The patient will be kept under our radar for surgical surveillance with annual CT chest scans.

TCACC constitutes a rare entity of thymic adenocarcinoma with limited available literature. The current data is derived from few case reports and case series. The histological overlap of these tumours and primary ACC of salivary glands poses a diagnostic challenge. Radiological investigations, immunohistochemical phenotyping and genetic analysis are crucial in establishing the diagnosis.

## Data Availability

All data and materials are available upon reasonable request from the corresponding author.
